# Amelioration of spinal cord injury in rats by blocking peroxynitrite/calpain activity

**DOI:** 10.1186/s12868-018-0450-z

**Published:** 2018-08-13

**Authors:** Mushfiquddin Khan, Tajinder S. Dhammu, Inderjit Singh, Avtar K. Singh

**Affiliations:** 1 Department of Pediatrics, 508 Children’s Research Institute, Medical University of South Carolina, 173 Ashley Ave, Charleston, SC 29425 USA; 2 Ralph H Johnson VA Medical Center, Charleston, SC USA; 3 Department of Pathology and Laboratory Medicine, Medical University of South Carolina, Charleston, SC USA

**Keywords:** BBB, Calpains, GSNO, nNOS, Peroxynitrite, SCI, Pain

## Abstract

**Background:**

Spinal cord injury (SCI) is one of the leading causes of disability and chronic pain. In SCI-induced pathology, homeostasis of the nitric oxide (NO) metabolome is lost. Major NO metabolites such as S-nitrosoglutathione (GSNO) and peroxynitrite are reported to play pivotal roles in regulating the activities of key cysteine proteases, calpains. While peroxynitrite (a metabolite of NO and superoxide) up regulates the activities of calpains leading to neurodegeneration, GSNO (a metabolite of NO and glutathione) down regulates the activities of calpains leading to neuroprotection. In this study, effect of GSNO on locomotor function and pain threshold and their relationship with the levels of peroxynitrite and the activity of calpain in the injured spinal cord were investigated using a 2-week rat model of contusion SCI.

**Results:**

SCI animals were initially treated with GSNO at 2 h after the injury followed by a once daily dose of GSNO for 14 days. Locomotor function was evaluated by “Basso Beattie and Bresnahan (BBB) locomotor rating scale” and pain by mechanical allodynia. Peroxynitrite level, as expression of 3-nitrotyrosine (3-NT), calpain activity, as the degradation products of calpain substrate alpha II spectrin, and nNOS activity, as the expression phospho nNOS, were measured by western blot analysis. Treatment with GSNO improved locomotor function and mitigated pain. The treatment also reduced the levels of peroxynitrite (3-NT) and decreased activity of calpains. Reduced levels of peroxynitrite resulted from the GSNO-mediated inhibition of aberrant activity of neuronal nitric oxide synthase (nNOS).

**Conclusions:**

The data indicates that higher levels of 3-NT and aberrant activities of nNOS and calpains correlated with SCI pathology and functional deficits. Treatment with GSNO improved locomotor function and mitigated mechanical allodynia acutely post-injury. Because GSNO shows potential to ameliorate experimental SCI, we discuss implications for GSNO therapy in clinical SCI research.

## Background

Spinal cord injury (SCI) results in locomotor deficits and pain due to the production of noxious metabolites which are held responsible for profound neurodegeneration [1, 2]. SCI is a major medical and socio-economic problem, and the rate of SCI is increasing every year [3]. The incidence of SCI is highest among young adults due to motor vehicle accidents, violence and sports accidents [4]. Other than critical care management, no current FDA-approved drug therapy exists for traumatic SCI [1]. Several pharmacological therapies, including methylprednisolone, have been evaluated time and again in SCI [2] without clinical success. SCI is divided into two distinct types of injury: primary and secondary. Primary (immediate phase after SCI) injury includes physical damage as a direct result of the traumatic event. It cannot be reversed. Secondary injury follows the initial physical insult, resulting from mechanistic crosstalk between and among several deleterious pathways, including redox and excitotoxicity [1]. Secondary injury is therefore amenable to reversal and treatment. A critical examination of injury mechanisms shows a disturbed nitric oxide (NO) metabolome [5, 6]. We hypothesize this metabolome to be responsible for the production of neuronal nitric oxide synthase (nNOS)-dependent deleterious peroxynitrite. As a consequence, much less NO is available for S-nitrosoglutathione (GSNO) biosynthesis and thus GSNO-mediated regulation of enzymatic activities is lost. Reduced NO bioavailability and the consequent decrease in GSNO levels are associated with chronic neurovascular injuries, and exogenous GSNO supplementation is reported to ameliorate CNS injuries [7–11]. Therefore, the objective of this study was to investigate the efficacy of GSNO for functional recovery and its role in regulation of the nNOS/calpain system in a rat model of contusion SCI.

GSNO is an endogenous molecule of the human body, produced mainly in NOS expressing cells by the reaction of NO with glutathione (GSH) in the presence of oxygen [12]. GSNO’s biosynthesis is also influenced by altered redox [13]. It is present in the brain and other organs [14]. GSNO reductase (GSNOR) is the major GSNO-degrading enzyme and thus GSNOR knock out mice store GSNO in excess [15]. GSNOR degrades GSNO to ammonia and oxidized glutathione (GSSG) without releasing free NO [16], indicating that the NO moiety of GSNO is not recycled by the enzymatic activity of GSNOR. GSNO is directly involved in cell signaling via S-nitrosylation of target proteins, including calpains, NF-κB, STAT3, neuronal NOS (nNOS) [8, 9, 17–21]. Several studies showing the efficacy of GSNO in human diseases have been listed by Hornyak et al. [22]. None of the studies reported significant side effects in humans associated with the use of exogenous GSNO. In animal studies, GSNO protects against cardiac ischemic injury [23], indicating the therapeutic potential of GSNO-mediated S-nitrosylation mechanism [9, 24]. S-nitrosylation of PTEN (a lipid phosphatase) has been shown to inhibit its activity, leading to the activation of Akt and thus the stimulation of neurorepair process in an animal model of stroke [25]. The Akt activation has been shown to be associated with stabilization of hypoxia-inducible factor-1 alpha (HIF-1α), which, in turn, induces the expression vascular endothelial growth factor (VEGF) leading to therapeutic angiogenesis/neurogenesis and consequent recovery of function [26].

In spite of the significant role of GSNO in cellular functions, neither GSNO nor its S-nitrosylation mechanism has been investigated for anti-neurodegenerative efficacy in SCI. Decreased synthesis of GSNO due to reduced levels of either GSH [19] or NO [27] or both in SCI, combined with increased degradation of GSNO by inflammation-induced enzyme activity of GSNO reductase (GSNOR) [24], will likely contribute to the reduced levels of GSNO in SCI. Deficient S-nitrosylation is considered to be a general neurodegenerative mechanism [28–30]. Via S-nitrosylation, GSNO protects against neurodegeneration by targeting multiple signaling pathways, including anti-inflammatory, anti-oxidant and vascular effects [9, 31–33]. GSNO also stimulates production of neurotrophic factors [11, 34] and induces neuroregeneration [35]. On the other hand, peroxynitrite is formed by an instantaneous diffusion limited reaction between NO and superoxide under oxidative conditions. This reaction not only reduces NO bioavailability but also increases peroxynitrite-mediated tissue/cell damage. Peroxynitrite causes a sustained activation of calpains [36], leading to neurodegeneration and functional deficits [10, 21]. In SCI, the observed increased 3-nitrotyrosine (3NT) levels, a peroxynitrite adduct of tyrosine residue, in the injured cord [37, 38] suggest its pathological role in SCI. We observed that GSNO treatment of SCI decreased the levels of peroxynitrite via inhibition of nNOS activation, which paralleled with decreased calpain activity and improved Basso Beattie and Bresnahan (BBB) locomotor rating scale scores as well as the threshold for mechanical allodynia out to 2 weeks post-injury.

## Methods

### Experimental procedure

#### Reagents

GSNO (Item#: GSNO-100) was purchased from World Precision Instruments (Sarasota, FL, USA). All other chemicals and reagents used were purchased from Sigma-Aldrich (St. Louis, MO), unless stated otherwise.

#### Animals

Animals were young adult male Sprague–Dawley (SD) rats, obtained from Harlan Laboratory (Wilmington, MA), weighing 250–300 g at the time of surgery. All animals received humane care in compliance with the Medical University of South Carolina’s (MUSC) guidance and the National Research Council’s criteria for humane care. Animal procedures were approved by the institutional animal care and use committee (IACUC) of MUSC.

#### Experimental groups, drugs and dose

The animals (n = 21) were randomly divided into three groups: (1) SCI animals treated with vehicle (SCI; n = 7), (2) SCI animals treated with GSNO (GSNO; n = 7), and (3) sham-operated treated with vehicle (Sham; n = 7). In the SCI + GSNO treatment group, the rats were administered freshly prepared GSNO (0.05 mg/kg body weight), which was dissolved in sterile saline (~ 25 μl) and administered iv at 2 h after SCI. The dose of GSNO treatment was based on our previously reported dose response curve study, using 10 µg to 100 µg/kg body weight in a rat model of SCI and TBI [7, 10, 39]. The dose 50 µg/kg was found most effective in reducing contusion volume measured at 7 days after TBI [39]. Tests on uninjured sham rats did not produce alterations in physiologic parameters (blood pressure, heart rate, and body temperature) measured at 1 h following GSNO treatment [39]. Details of a GSNO study on physiologic parameters in rats have been previously described [10, 19].

#### Rat model of contusion SCI

Surgical anesthesia was induced by ketamine (90 mg/kg body weight) and xylazine (10 mg/kg body weight) administered intraperitoneally (ip). The animal was then placed onto a heated pad, and core body temperature was maintained at 37.0 ± 1 °C. The animals were secured in a stereotaxic frame. A dab of sterile ophthalmic ointment was placed on each eye to compensate for the decrease in lacrimation during anesthesia. SCI at the T9-T10 level was produced on the exposed spinal cord following a dorsal median incision and laminectomy. SCI was induced using a computer controlled impactor device described by Dr. Bilgen [40] and used in our studies [7, 41, 42] under aseptic conditions. SCI was performed with 2 mm tissue deformation and an impact velocity of 1.5 m/s and contusion time 85 ms. These parameters and conditions produced reproducible moderate spinal cord injury as described in our publications [7, 41]. Sham animals had the same procedures, with the exception of the impact. The impact tip was wiped clean with sterile alcohol after each impact and cleaned/disinfected further with cidex after surgery. During impact, body temperature was maintained at 37 °C by a heating pad. Immediately after injury, the incision was closed with nylon suture, and 2% lidocaine jelly was applied to the lesion site to minimize any possible discomfort. Post-surgical care: the bladders of all animals were expressed two to three times per day initially and later as needed. The body weight and humane endpoints were regularly monitored. Analgesic treatment was avoided after surgery because pain is also a target of this investigation. Antibiotic treatment was used in the event of persistent infection, which occurred rarely. The animals were sacrificed after the specified period of time with an overdose of ketamine/xylazine (90/10 mg/kg body weight) administered ip, as approved by the IACUC of MUSC.

#### Evaluation of locomotor function

All 7 rats were assessed at the indicated time points using the “Basso Beattie Bresnahan (BBB) locomotor rating scale” [43]. The BBB rating was described with a 21-point scale to measure hind limb function at various time points after injury. The scale assesses several different categories, including limb movement and tail position [43]. In our experiments, sham operated animals scored 21 (normal) on the BBB rating scale, whereas the SCI animals at day 0 had complete hind limb paralysis, thus scoring 0. Two investigators blinded to the experimental groups evaluated rats using the BBB scale as previously described from our laboratory [7]. All rats in both SCI and GSNO (SCI + GSNO) had significantly lower BBB score evaluated on day 1 after SCI.

#### Evaluation of mechanical allodynia

Before the testing of mechanical allodynia, all rats were habituated for at least 2 h on a metal mesh inside a *von Frey* plastic chamber. Nociception was measured by the paw pressure threshold using anesthesiometer (AM) (Ugo Basile, Italy), which applies a linearly increasing mechanical force to the dorsum of the rat’s hind paw. The test was performed as previously described from our laboratory [44, 45]. The nociceptive threshold was defined as the force in grams at which the rat withdrew its paw. Continuously increasing pressure was applied to the dorsal surface of the hind paws. The time the animal withdrew its paw was recorded. Three trials were made on each paw with 5 min inter-test intervals. Testing was performed once per day until the end of the experiment. All rats in both SCI and GSNO (SCI + GSNO) developed significant pain when evaluated on day 3 after SCI.

#### Western blot analysis

At the endpoint, the animals were euthanized by decapitation under deep anesthesia and spinal cord was harvested for biochemical experiments. The spinal cords were snap frozen and stored at − 70 °C for subsequent assays, if needed.

In the traumatic penumbra area (8 mm segment consists of 2 mm epicenter, 3 mm caudal from epicenter, 3 mm rostral from epicenter) from the injured cord tissue, western blot was performed as described earlier [9, 46] using following antibodies. nNOS (Abcam Cat# ab1376, RRID:AB_300614, 0.2 µg/ml concentration), phospho nNOS Ser^1417^, equivalent to human Ser^1412^ (Abcam Cat# ab90443, RRID:AB_2049208, 1.0 µg/ml concentration), 3-NT (Abcam Cat# ab7048, RRID:AB_305725, 0.1 µg/ml concentration), alpha II spectrin (Cell Signaling Cat# SC-46696, RRID:AB_671135, 0.2 µg/ml concentration) and β-actin (Sigma-Aldrich Cat# A3853, RRID:AB_262137, 0.2 µg/ml concentration), followed by horseradish peroxidase-conjugated, goat anti-rabbit secondary antibody (Jackson ImmunoResearch Lab Cat# 111-035-045, RRID:AB_2337938, 1:4000 dilution). All non-phospho antibodies were diluted with 1XTBS-T with 2% non-fat dry milk. The pnNOS antibody was diluted using 1X TBS-T with 2% bovine serum albumin. Protein concentrations were determined using protein assay dye from Bio-Rad Laboratories (Hercules, CA). Twenty microgram protein was used for western analysis. Densitometry of protein expression was performed using a GS800 calibrated densitometer from Bio-Rad laboratories (Hercules, CA).

#### Statistical evaluation

Statistical analysis was performed using software Graph pad Prism 5.01 as described previously [35]. The results are presented as the mean ± SD. Statistical significance was analyzed by one-way or two-way (ANOVA) with repeated measures with time, and Bonferroni post hoc test was used for multiple comparisons. A *p* value < 0.05 was considered significant.

## Results

### Effects of exogenous GSNO treatment on locomotor function

**Fig. 1:**
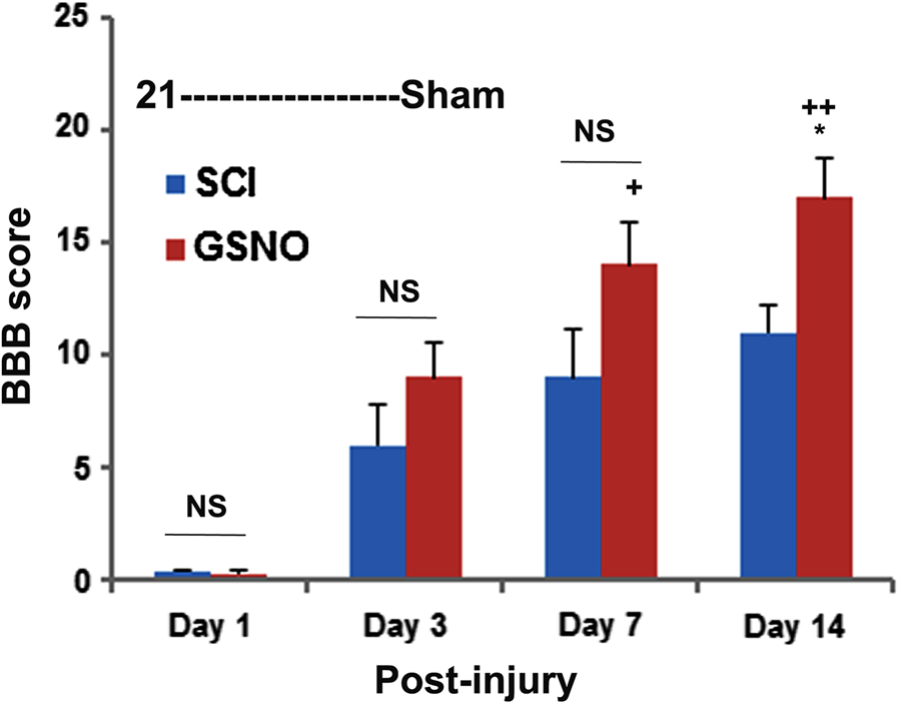
Effect of GSNO on locomotor function. Studies on locomotor function using BBB locomotor rating scale were performed at days 1, 3, 7 and 14. BBB rating was evaluated by a blinded observer. Score of 21 (BBB) was assigned to sham animals displaying coordinated gait, consistent toe clearance, lifted tail, steady trunk, and parallel paw position throughout their stance. Data are presented as mean ± standard deviation (n = 7). ^+^*p* < 0.05 versus GSNO day 1, 3, ^++^*p* < 0.01 versus GSNO day 1, 3, *p < 0.05 versus SCI day 14. *NS* non-significant

Evaluation of locomotor functions using BBB score in rats is the standard method [43, 47, 48] to determine the efficacy of a preclinical/test drug in SCI. BBB scoring at 1, 3, 7 and 14 days shows that SCI rats had significantly greater impaired motor function compared with sham animals (Fig. 1). GSNO treatment significantly improved the recovery of locomotor function on day 14 (*p* < 0.001) compared with the SCI group animals (Fig. 1). The data showed slow but steady recovery with time, supporting the efficacy of GSNO for functional improvement following SCI.

### Effects of exogenous GSNO treatment on neuropathic pain

**Fig. 2:**
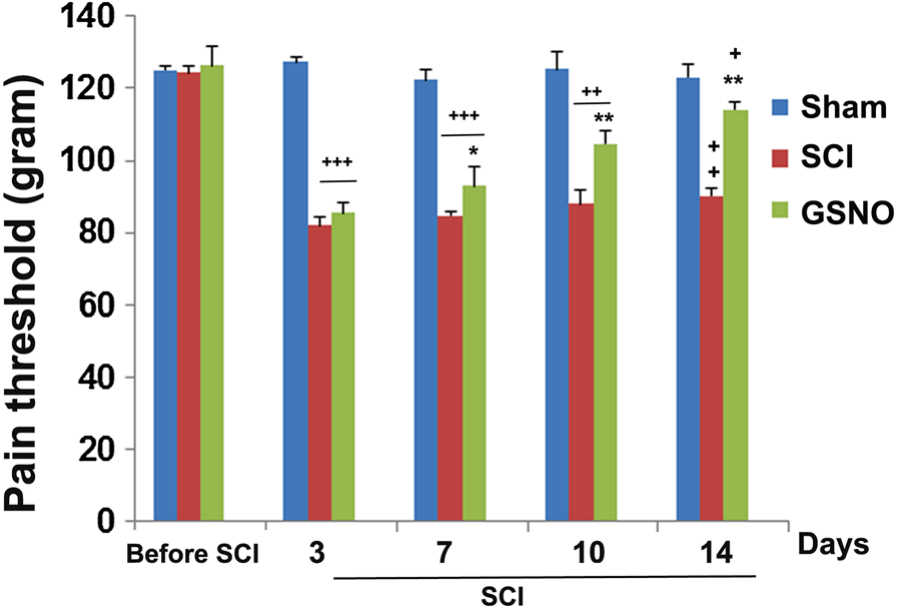
Effect of GSNO on nociception. Pain threshold was measured with aesthesiometer (AM) for 14 days following SCI. GSNO treatment significantly improved the hyperalgesia associated with SCI. Data are presented as mean ± standard deviation (n = 7). ^+++^*p* < 0.001; ^++^*p* < 0.01; ^+^*p* < 0.05 versus Sham, **p < 0.01; *p < 0.05 versus SCI

Chronic neuropathic pain is associated with SCI, with substantial impact on quality of life in humans [49, 50]. Significant mechanical sensitivity differences were observed in both SCI and GSNO-treated SCI (GSNO) groups after SCI compared with the sham group. From day 7 onward, the GSNO group had a significantly improved/increased mechanical withdrawal latency compared with the SCI group (Fig. 2), indicating an improved pain threshold.

### Effects of exogenous GSNO treatment on the levels of peroxynitrite (3-NT) and the activation of nNOS (Ser^1412^ phosphorylation)

**Fig. 3:**
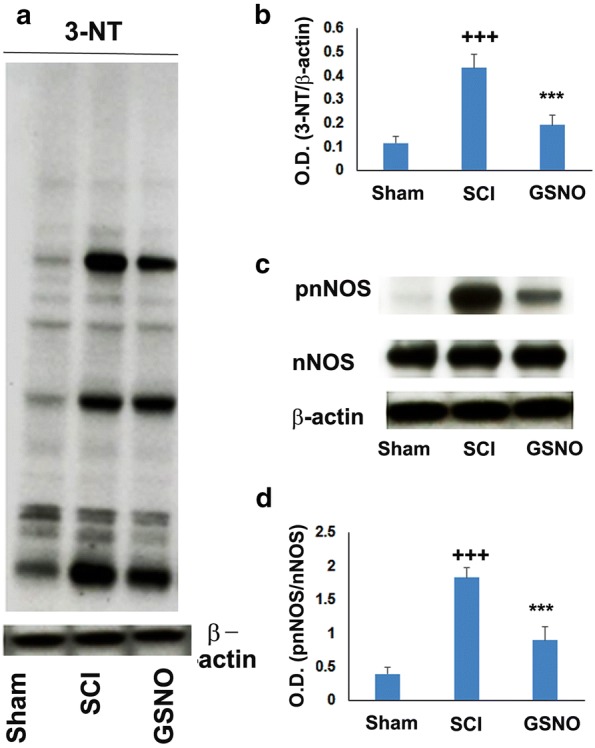
Immunoblots of 3-NT, nNOS, and phosphorylated nNOS (Ser^1412^) in the traumatic penumbra (immediately after epicenter) at 14 days after SCI. SCI increased the expression levels of 3-NT (**a**), its densitometry (**b**), phosphorylated nNOS (Ser^1412^) (**c**), and its densitometry (**d**). GSNO treatment of SCI decreased expression levels (**a**–**d**). Expression of nNOS remained unchanged in all three groups (**c**). Data are presented as mean ± standard deviation (n = 7). ^+++^*p* < 0.001 versus Sham and ***p < 0.001 versus SCI

We and others have identified neuronal peroxynitrite as a major causative factor in SCI pathology [37, 38, 46, 51], and decreasing peroxynitrite levels by GSNO is a major mechanism in GSNO-mediated neuroprotection and functional recovery in TBI [8, 10, 39]. Neuronal peroxynitrite is produced by the aberrant activity of nNOS after CNS trauma. Peroxynitrite levels, measured by the expression of 3-NT, were significantly higher in the SCI compared with the sham group (Fig. 3a, b, *p* < 0.001). Treatment with GSNO significantly decreased these elevations (Fig. 3a, b). The levels of 3-NT in SCI correlated well with the activation of nNOS (increased phosphorylation at Ser^1412^) compared with the sham group (Fig. 3c, d, *p* < 0.001). GSNO treatment of SCI significantly down regulated nNOS activation compared with SCI (Fig. 3c, d, *p* < 0.001). The parallel between the levels of 3-NT and the activation of nNOS (pnNOS) indicates that SCI-induced peroxynitrite may have originated mainly from nNOS, and the activity of nNOS is regulated/inhibited by GSNO, likely via S-nitrosylation.

### Effects of exogenous GSNO treatment on the activity of calpains measured as α-II-spectrin breakdown products

**Fig. 4:**
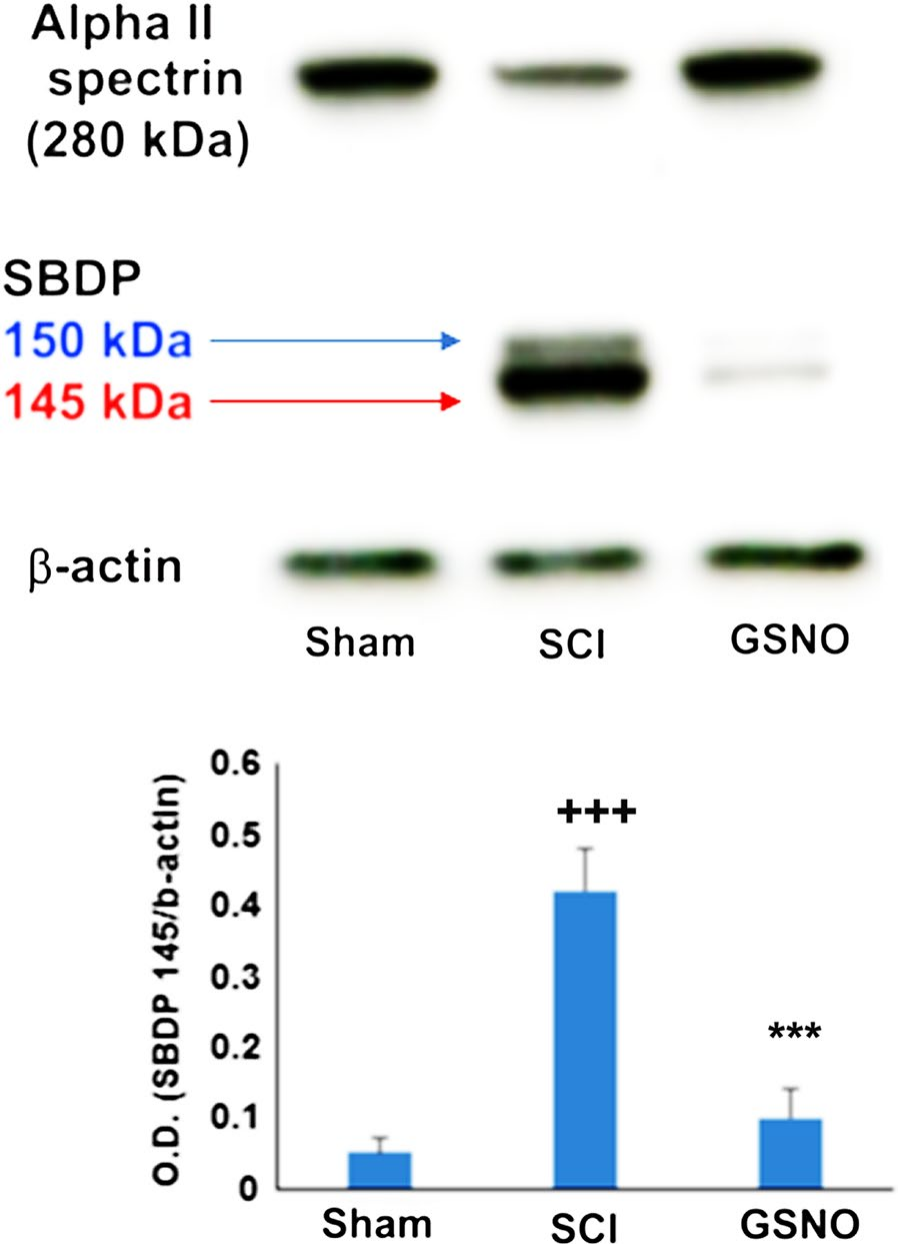
Immunoblots of α-II-spectrin in the traumatic penumbra at 14 days after SCI. SCI increased α-II-spectrin breakdown products (SBDP 145 kDa, indicated by red arrow) (**a**) and its densitometry (**b**). GSNO treatment of SCI decreased SBDP 145 kDa (**a**, **b**). Data are presented as mean ± standard deviation (n = 7). ^+++^*p* < 0.001 versus Sham, ***p < 0.001 versus SCI

Neuronal alpha-II-spectrin (280 kDa) is one of the major substrates of calpains [52]. The calpain-specific alpha II spectrin breakdown product (SBDP)145 kDa fragment is used as a marker of calpain activity [53]. The band at 150 kDa is also a cleavage product of calpain activity; however, it is not specific to calpain activity [54]. The intensity of the 150 kDa band was significantly less than the 145 kDa band. Calpain activity, measured via α-II-spectrin breakdown product (SBDP) 145 kDa, was significantly higher (*p* < 0.001) in the SCI group compared with the sham (Fig. 4a, b). GSNO treatment of SCI significantly (*p* < 0.001) decreased the levels of SBDP 145 kDa, indicating that GSNO decreased the activity of calpains (Fig. 4a, b). The activity of calpains (Fig. 4) correlated well with levels of 3-NT and the activation of nNOS, as shown in Fig. 3.

## Discussion

This is a preliminary mechanism-based study showing that SCI-induced functional deficits (Fig. 1), and neuropathic pain (Fig. 2) paralleled aberrant activation of nNOS, increased levels of peroxynitrite (Fig. 3) and high activity of calpains (Fig. 4) in a 2-week rat model of contusion SCI. The study further shows the therapeutic efficacy of GSNO. It improved functional deficits (Fig. 1) and increased the pain threshold (Fig. 2) by inhibiting the activities of both nNOS (Fig. 3) and calpains (Fig. 4) and reducing the levels of injurious peroxynitrite (Fig. 3).

Locomotor function deficits and pain are two major consequences intrinsic to SCI [49]. Deleterious metabolites, formed by the aberrant activities of otherwise regulatory enzymes such as nNOS, are primarily responsible for producing potent oxidizing/neurodegenerating agents, such as peroxynitrite, in neurons. Excessive accumulation of neuronal peroxynitrite is implicated in neuronal cell death and subsequent neurodegeneration [55]. In fact, scavenging peroxynitrite using peroxynitrite decomposition catalysts such as FeTPPS has been reported to ameliorate SCI [56], supporting this direct deleterious role of peroxynitrite. Inhibition of nNOS activity following SCI [57] has also been shown to provide neuroprotection, and nNOS KO mice show improved recovery after SCI [58], indicating a deleterious role of nNOS activity in SCI. An nNOS-based therapy for SCI therefore offers a logical approach. Reversible down regulation of nNOS activity, such as via-S-nitrosylation, is preferred because it maintains the required physiological activity of nNOS. The roles of other NOS enzymes (inducible and endothelial) in the chronic phase pathology, such as in neurodegeneration and pain, is not clear [59]. Peroxynitrite produced in neurons is a product of an instantaneous reaction between nNOS-derived NO and superoxide. Because NO is used for the formation of peroxynitrite (3-NT), the biosynthesis of GSNO, a product of a slow reaction between NO and GSH, and GSNO-mediated regulatory mechanisms are derailed. Because deleterious nNOS activity is down regulated by a GSNO-mediated S-nitrosylation mechanism [60], reduced NO/GSNO levels contribute to nNOS-dependent neurodegeneration and pain SCI pathology. Such a derailed NO/GSNO metabolism in SCI may also be responsible for functional deficits. Therefore, we tested the hypothesis that GSNO reduces the levels of peroxynitrite, inhibits the activity of nNOS, and improves behavioral function and cellular plasticity in young adult male rats.

Pain is one of the major issues in SCI for obvious reasons but also because it impairs recovery after SCI [61]. Both inflammatory and neuropathic pain (caused by a lesion or disease of somatosensory function) are present in the majority of SCI patients [50, 62]. Due to the lack of mechanistic understanding of pain, satisfactory pain-management therapy of SCI is not yet available. We observed that the GSNO treatment significantly increased the pain threshold and reduced calpain activity after 2 weeks of SCI (Fig. 2), indicating that GSNO possesses an analgesic property in addition to improving functional deficits (Fig. 1). Furthermore, significant increases in tissue peroxynitrite levels (Fig. 3) correlated well with calpain-mediated cytoskeleton degradation (Fig. 4), indicating peroxynitrite’s contribution to neurodegeneration. Recently, we have shown that the activity of calpains is upregulated by peroxynitrite whereas GSNO, via S-nitrosylation, inhibits the activity of calpains in TBI [8], indicating a similar role of peroxynitrite versus GSNO in this SCI study. Peroxynitrite originating from nNOS [63] and NMDA receptor activity [64] is also recognized among the prominent causes of neuropathic pain following nerve injury. GSNO, likely via S-nitrosylation, down regulates the activity of nNOS, thus reducing the levels of peroxynitrite and its associated pain. Decreased levels of peroxynitrite in brains and improved neurological functions have also been shown after GSNO treatment in rat models of stroke and TBI [34, 51], indicating that the mechanism of S-nitrosylation invokes anti-neurodegeneration and anti-pain activities in CNS trauma. These observations establish the therapeutic potential of GSNO-mediated mechanisms in simultaneously treating neurodegeneration and neuropathic pain following SCI.

Inflammation is another significant component of SCI, contributing to neurodegeneration and pain. Interestingly, GSNO-mediated mechanisms are also shown to down regulate the expression of pro-inflammatory cytokines and NF-κB [18, 39], as well as the activation of STAT3 [20]. These actions contribute to the reduction of inflammation-mediated neurodegeneration and pain. As an alternative mechanism to alleviate pain in SCI, IL-10 has been shown to be a potent anti-neuropathic pain molecule [65–67], and GSNO-mediated mechanisms are reported to upregulate the levels of IL-10 [68] as well as to reduce pain in a rat model of cauda equine compression [44]. We add one caveat that excessive accumulation of GSNO, as observed in GSNOR knock out mice, creates altered redox pathology, leading to sensitization to pain [15] and thus rendering GSNOR knock out mice ‘not suitable’ for pain related studies. A critical balance of NO/GSNO versus peroxynitrite is requisite to maintain the homeostasis of the NO metabolome, ameliorating SCI pathology. Low dose exogenous supplementation of GSNO seems to be an ideal approach to improve pain threshold and to provide neuroprotection. One advantage of using GSNO supplementation is that GSNO-mediated regulatory mechanisms are reversible, and thus the physiological levels of activity of targeted enzymes can be maintained. The improved BBB score (Fig. 1) and increased pain threshold (Fig. 2) reported here support the efficacy of GSNO in ameliorating SCI and provide a rationale to further investigate GSNO therapy in SCI. *Limitations* the most affected population from SCI is young adult males. Therefore, we used young adult male animals in this preliminary study; however, the exclusion of female rats demands further testing in these populations. We are also aware that a 2-week SCI study is relatively short to sufficiently characterize the injury course. Moreover, biochemical studies of the early acute phase are needed. Therefore, in follow-up study, the efficacy of GSNO therapy and the cause-and-effect relationship between GSNO and peroxynitrite/calpain system will be investigated for both acute and longer chronic periods of time, using both male and female young adult animals.

## Conclusions

Under SCI pathology, superoxide reacts with NO and this reaction produces a large amount of injurious peroxynitrite. Increased levels of peroxynitrite cause an upregulation of calpain activity and thus neuronal cytoskeleton degradation. Peroxynitrite is also recognized as a mediator of pain. Under such pathological conditions, S-nitrosylation-mediated biological regulation (inhibition) of the activity of nNOS and calpain is lost due to its reduced bioavailability, and thus levels of NO/GSNO. Replenishment of exogenous GSNO was found to inhibit the activation of nNOS, thus blocking the production of peroxynitrite and reducing the activity of calpains, leading to improved locomotor function and decreased mechanical allodynia acutely post-injury in SCI animals. Furthermore GSNO’s administration to humans is not associated with adverse effects [22]. Therefore, testing the efficacy of exogenous GSNO in humans may lead to an SCI therapy of clinical relevance.

## Abbreviations

BBB: Basso Beattie and Bresnahan
GSNO: S-nitrosoglutathione
HIF-1α: hypoxia-inducible factor-1 alpha
IHC: immunohistochemistry
nNOS: neuronal nitric oxide synthase
NO: nitric oxide
3-NT: 3-nitrotyrosine
PTEN: phosphatase with sequence homology to tensin
SCI: spinal cord injury
Sham: sham-operated animals
VEGF: vascular endothelial growth factor
